# Impact of phytosterols on liver and distal colon metabolome in experimental murine colitis model: an explorative study

**DOI:** 10.1080/14756366.2019.1611802

**Published:** 2019-05-10

**Authors:** Nunzia Iaccarino, Jussara Amato, Bruno Pagano, Anna Di Porzio, Matteo Micucci, Luca Bolelli, Rita Aldini, Ettore Novellino, Roberta Budriesi, Antonio Randazzo

**Affiliations:** a Department of Pharmacy, University of Naples Federico II, Naples, Italy;; b Department of Pharmacy and Biotechnology, University of Bologna, Bologna, Italy

**Keywords:** Phytosterols, ulcerative colitis, GC-MS, metabolomics, multivariate data analysis

## Abstract

Phytosterols are known to reduce plasma cholesterol levels and thereby reduce cardiovascular risk. Studies conducted on human and animal models have demonstrated that these compounds have also anti-inflammatory effects. Recently, an experimental colitis model (dextran sulphate sodium-induced) has shown that pre-treatment with phytosterols decreases infiltration of inflammatory cells and accelerates mucosal healing. This study aims to understand the mechanism underlying the colitis by analysing the end-products of the metabolism in distal colon and liver excised from the same mice used in the previous work. In particular, an unsupervised gas chromatography-mass spectrometry (GC-MS) and NMR based metabolomics approach was employed to identify the metabolic pathways perturbed by the dextran sodium sulphate (DSS) insult (i.e. Krebs cycle, carbohydrate, amino acids, and nucleotide metabolism). Interestingly, phytosterols were able to restore the homeostatic equilibrium of the hepatic and colonic metabolome.

## Introduction

Inflammatory bowel disease (IBD) is a group of chronic inflammatory conditions affecting the gastrointestinal tract^1^. Ulcerative colitis (UC) and Crohn’s disease (CD), which represent the most common IBD subtypes, are both characterised by a dysregulated immune response to commensal flora caused by genetic, environmental, and microbiological factors. However, the accurate aetiology of IBD is still not completely understood^2^^,^^3^. Even though, the highest incidence rate of IBD has traditionally been reported in North America and Western Europe, a very recent study reports that its occurrence is increasing in newly industrialised countries in Africa, Asia, and South America, including Brazil and Taiwan, whose societies have become more westernised. These data highlight the importance of research for prevention of IBD to manage this complex and costly disease^4^.

Phytosterols are plant derived sterols, structurally related to cholesterol. They have been classified into (i) sterols, which have a double bond in the sterol ring, and (ii) stanols, which conversely lack a double bond in the sterol ring, thus being saturated molecules. Phytosterols are known for their hypocholesterolaemic^5^ and anti-inflammatory properties^6^^,^^7^ that are independent from each other^8^. Among the anti-inflammatory studies, a discrete number of investigations have been conducted in the last years to understand their role on IBD. Experimental IBD models are often used for this purpose where the trinitrobenzene sulphonic acid (TNBS) or dextran sodium sulphate (DSS) is employed to generate models that mime respectively CD and UC. IBD animal models are often chosen for their robustness and resemblance to human IBD regarding the disruption of the colonic epithelial layer and the progressive immune and inflammatory responses^9^^,^^10^. In this frame, Aldini et al. performed for the first time a longitudinal preventive and therapeutic study about the role of phytosterols in the prevention/induction and remission of inflammation in an experimental murine colitis model^11^. In particular, a commercially available nutraceutical, based on a mixture of β-sitosterol, campesterol, stigmasterol, and brassicasterol (Supplementary Figure S1), was employed in mice with DSS-induced colitis as well as in healthy mice. The results of such study showed that pre-treatment with phytosterols reduces the clinical symptoms and exerts a protective effect on the induced colonic inflammation, decreasing infiltration of inflammatory cells, and accelerating mucosal healing. These effects have been related to the phytosterols antioxidant properties, thus suggesting that they may actually be taken in consideration as potential nutraceutical tool in the management (prevention/remission) of IBD and other intestinal inflammatory diseases.

From the above study, it was clear that phytosterols play an important role in restoring the initial structural and biochemical environment in the gastrointestinal tract of the colitis mice. In order to better understand the comprehensive metabolic perturbation induced by the phytosterols administration both in colitis and control mice; we decided to undertake a metabolomic study on the same mice employed by Aldini et al. The aim of a metabolomic study was to provide a detailed analysis of the metabolites (substrates and products) in metabolic pathways that are altered by a perturbation^12^. Currently, there are two major analytical techniques employed in the metabolomic field: nuclear magnetic resonance (NMR) spectroscopy and mass spectrometry (MS)^13–16^. Both techniques have inherent advantages and disadvantages that can influence the analytical coverage of the metabolome. Thus, when possible, a combined application of NMR spectroscopy and MS definitively represents the best approach to get a complete picture of the metabolome under study^17^. The importance of a metabolomic approach to evaluate the impact of IBD pathologies in humans^18–23^ and animal models^24–27^ has been widely proven in the literature. These studies revealed that specific metabolites, associated with gut microbiota, oxidative stress, and regulation of energy levels, were significantly altered by DSS insult. In addition, Karlsson et al.^28^ found that the DSS-induced colitis causes major alterations in hepatic fatty acids metabolism resembling human IBD, suggesting that the model can also be used for target discovery and validation of hepatic-related metabolic alterations. In this frame, we decided to focus our attention both on the colon (where the inflammation damage is located) as well as on the liver (hub of metabolism) of mice, by employing a combined application of gas chromatography-mass spectrometry (GC-MS) and NMR based metabolomics to evaluate the effect of the phytosterol mixture employed.

## Materials and methods

### Chemicals

Analytical grade chloroform, methanol, trimethylsilyl cyanide (TMSCN) (99.8%) and C10–C40 all-even alkane mixture were purchased from Sigma-Aldrich (St. Louis, MO). Water used throughout the study was purified using a Millipore Milli-Q lab water system (Merck Millipore Corporation, Merck KGaA, Darmstadt, Germany) equipped with a 0.22 µm filter membrane. Administered phytosterols were supplied by Solgar (Solgar Italia^®^ Multinutrient^®^ S.p.A., Padova, Italy). The detailed composition of the nutraceutical preparation is reported in Supplementary Table S1.

### Animal study

**Figure 1.**
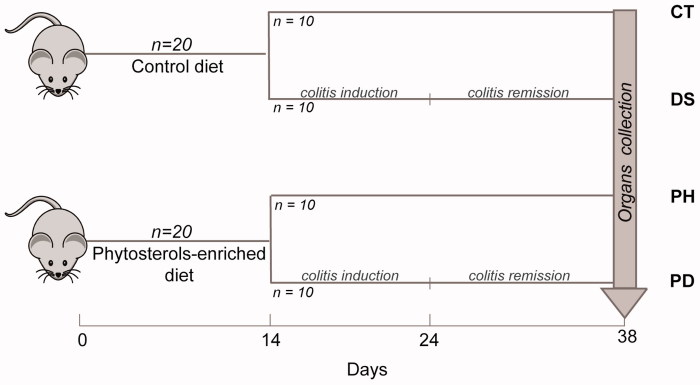
Overview of the study design. A total of 40 male Balb/c mice were used for the trial. Twenty mice received a control diet and, after 14 days, were split into two subgroups: Controls (CT) that continued to receive the same diet, and DSS treated group (DS) that received water containing DSS *ad libitum* in order to induce the colitis. The same procedure was employed for the mice fed the phytosterols-enriched diet.

Forty male Balb/c mice (8 weeks old) weighing from 22 to 25 g (Charles Rivers Laboratories, Calco, Italy) were enrolled. A detailed description of the intervention, dietary composition, and phytosterols mixture can be found in the paper by Aldini et al.^11^ As shown in Figure 1, the mice were divided into two groups in the first day of the study; half of the animals received the usual commercial control diet, while the other half were fed the same diet enriched with phytosterols preparation. After 14 d, the group fed the control diet was split into two subgroups: controls (CT), which continued to receive the same diet, and DSS treated group (DS) that received drinking water *ad libitum* containing DSS (MP Biomedicals, Solon, OH; molecular weight 36,000–50,000) 5% (v/w) until day 24. Acute colitis was induced by DSS^29^. At the same time, also the group fed the phytosterol-containing diet was split into two subgroups, following exactly the same procedure described above. Thus, the phytosterols-treated group (PH) and the colitis phytosterol-fed group (PD) were generated. The mice of four groups were sacrificed at the end of the experiment and liver and distal colons were recovered. The organs were extracted and subjected to a metabolomic study in order to investigate the effect of a phytosterols mixture on the treated mice. Both liver and distal colon samples were analysed by GC-MS, while only the liver samples (more abundant) were further investigated by NMR. Twenty-four hours before the experiments, food supply was withdrawn, while water was maintained *ad libitum*. The animals were sacrificed by cervical dislocation. The collection of the organs was obtained at the end of the remission period, in order to evaluate a steady state of the colitis follow-up period. A liver and colon sample, for each mouse, were collected and lyophilised. Briefly, the tissue samples were frozen in Eppendorf vials at −20 °C for almost 1 h. The vials were transferred in the freeze-dryer chamber of an Edwards Minifast 680 (Norfolk, England) cooled at −20 °C and lyophilised in two steps: a low temperature ramp from −20 to 4 °C during 12 h and a steady temperature of 20 °C since the pressure reached 4 × 10^−2^ mbar. After lyophilisation, the chamber pressure rebalance was done using nitrogen gas.

### Ethical statement

The work has been conducted according to the guidelines set forth by EEC Directive 86/609 on the care and use of experimental animals. The protocol for the induction of colitis was approved by the Institutional Ethics Committee of the University of Bologna (Protocol 22/03/10). All studies involving animals are reported in accordance with the ARRIVE guidelines (https://www.nc3rs.org.uk/arrive-guidelines). Animals were housed in a controlled environment (22–24 °C), maintained on a standard 12-h light/dark cycle (lights on at 07.00 h), and had free access to food and water throughout the study. The authors confirm that the protocol was specifically approved by the Institutional Animal Care and Use Committee (“Comitato Etico Scientifico per la Sperimentazione Animale”) of the University of Bologna and transmitted to the Ministry of Health (art. 7–8–9 D. Lgs.116/92).

### Metabolite extraction

A dual phase extraction procedure was performed on colon and liver samples. Basically, a mixture of water, methanol, and chloroform in the volume ratio of 1.8:2:2 was added to the lyophilised samples^30^^,^^31^. About 40 mg of liver and 8 mg of distal colon were collected and mixed with about 960 and 800 µL, respectively, of extracting mixture. Then, a homogenising step was needed to break up the tissues and let the partition of the metabolites in the extracting solvents. A FastPrep-24^®^ (MP Biomedicals, Eschwege, Germany) tissue homogeniser with specialised Lysing Matrix beads was employed for this purpose. After this step, the supernatant was separated from the pellet, which was discarded. The supernatant was centrifuged at 15,000 rpm at 4 °C for 15 min. This procedure generated a two-phase extract: the aqueous upper phase containing hydrophilic metabolites, while apolar metabolites as lipid molecules moved in the organic lower phase. Proteins and macromolecules were trapped, instead, in the thin skin-like layer between the two phases. The upper and lower phases were separated and transferred into different tubes. Finally, solvents were completely removed from both fractions using a vacuum concentrator. Only the hydrophilic phase was considered in this study.

### GC-MS-TOF data acquisition

A total of 40 µL of final metabolite extract of each sample were put in a glass insert, dried first in a SpeedVac Concentrator (Thermo Fisher Scientific Co., Waltham, MA), for 4 h, and then put in the lyophiliser, overnight. The glass inserts were sealed with air tight magnetic lids into GC-MS vials and derivatised by addition of 40 µL of TMSCN as described, elsewhere^32^. Derivatisation and injection were fully automated using a PAL autosampler (AS) with Robotic Tool Change system (CTC Analytics, Zwingen, Switzerland) integrated to the GC-MS-TOF (Pegasus BT, LECO Corporation, Saint Joseph, MI). The GC-MS consisted of an Agilent 7890B GC (Agilent Technologies, Santa Clara, CA) and a time-of-flight mass spectrometer (LECO Corporation, Saint Joseph, MI). After the addition of the derivatisation reagent, samples were transferred into the agitator of the AS and incubated at 40 °C for 40 min at 750 rpm. This procedure ensures precise derivatisation time and reproducible sample injections. Immediately after derivatisation, 1 µL of the derivatied sample was injected in splitless mode into the injection port. The septum purge flow and purge flow were set to 25 and 15 mL/min, respectively. The injection port temperature was set to 250 °C. GC separation was performed on a Rxi-5MS 5% Phenyl 95% Dimethylpolysiloxane column (30 m with I.D. 0.25 mm and film thickness 0.25 µm) (Restek, Bellefonte, PA). The initial temperature of the GC oven was set to 40 °C and held for 2 min, followed by heating at 12–320 °C min^−1^ and kept for an additional 8 min, making the total run time 33.3 min. Mass spectra were recorded in the range of 45–600 *m/z* with an acquisition rate of 10 spectra/s, and MS detector and ion source were switched off during the first 6.4 min of solvent delay time. The transfer line and ion source temperature were set to 280 and 250 °C, respectively. Helium (grade 6.0) was used as carrier gas, at a constant flow rate of 1 mL/min. The mass spectrometer was tuned according to manufacturer’s recommendation using perfluorotributylamine (PFTBA). The AS and GC-MS were controlled using vendor software PAL Sample Control (CTC Analytics, Zwingen, Switzerland) and ChromaTOF (LECO Corporation, Saint Joseph, MI), respectively. Two technical replicates were prepared for each sample (for a total of 80 samples); they were randomised prior to derivatisation and GC–MS analysis. In order to monitor the instrument performance, a blank sample containing only derivatisation reagent, a quality control (QC) sample (pooled sample), and an alkane mixture standard sample (all even C10–C40 alkanes at 50 mgL^−1^ in hexane) were injected after every 10 real samples. The raw GC-TOF-MS data was processed by the ChromaTOF software version 5.03.09.0 (LECO Corporation, Saint Joseph, MI) that deconvolutes mass spectra and performs peak identification using NIST11 library (NIST, Gaithersburg, MD). The library search was set to return top 10 hits with EI-MS match of >80% using normal-forward search and with a mass threshold of 20. Deconvoluted peaks were aligned across all samples considering a retention time shift allowance of <2 s and the presence of each metabolite in >80% of all pooled control samples. In this way, final datasets consisting of 53 metabolites for the liver and 32 metabolites for the colon samples were obtained. The identification computed by the ChromaTOF was classified according to the levels indicated by the Metabolomics Standard Initiatives (MSI)^33^, (i) Level 1 if the peaks are confirmed using authentic standards, (ii) Level 2 when the peaks are identified based on their EI-MS match ≥80 (%) and retention index (RI) match (±30), and (iii) Level 3 when the peaks are identified based on their EI-MS match ≥65 (%). In our case, no authentic standards were employed and only the metabolites characterised by EI-MS match ≥80 (%) were considered for data analysis. Thus, in this work, Level 3 includes the metabolites that have EI-MS match ≥80 (%) but largely differ in RI from the relative compound available in the NIST library. The detailed list of identified metabolites is reported in Supplementary Table S2.

### NMR data acquisition

For quantitative reasons, only the liver samples could be analysed by NMR. All dried polar extracts were resuspended in 540 µL of D_2_O together with 60 µL of 1 M sodium phosphate buffer (pH 7.2) to give a final buffer concentration of 0.1 M. Samples were vortexed briefly and transferred into 5 mm NMR tubes for analysis. One-dimensional ^1^H-NMR spectra were acquired at 37 °C, employing a 500 MHz Varian Unity Inova spectrometer (Lubbock, TX) equipped with a 5 mm ^1^H{^13^C/^15^N} triple resonance probe. ^1^H-NMR spectra of hydrophilic cell extracts were acquired using a pulse sequence that includes presaturation in order to suppress the water signal. All the experiments were acquired with a relaxation time of 3 s, a pulse width of 3.83 µs, an acquisition time of 1.5 s, 256 scans, and a spectral width of 6999.7 Hz. The assignment of the main hydrophilic metabolites was done according to the literature^34^^,^^35^. Chenomx NMR suite software (Chenomx Inc., Edmonton, Canada) and the Human Metabolome Database (HMDB) (University of Alberta, Edmonton, Canada) helped the assignment. The NMR spectra were processed using iNMR (www.inmr.net) software and then imported in Matlab (R2015b The Mathworks Inc., Natick, MA). The NMR regions above 9.5 ppm and below 0 ppm were removed because containing only noise. Furthermore, regions between 7.67 and 7.72 ppm and between 3.35 and 3.38 ppm were discarded because of the residual signals of chloroform and methanol (employed during the extraction procedure), respectively. Finally, the region between 4.56 and 5.06 was discarded because of the residual water signal. A global alignment step was carried out using icoshift^36^ and choosing the acetate singlet at 1.92 ppm as reference signal.

### Data analysis

Data generated from the GC-TOF-MS, were analysed using both univariate (ANOVA) and multivariate, such as principal component analysis (PCA)^37^ approaches. Before the data analysis, some pre-processing steps were performed. To minimise non-sample related variations, the final metabolite matrix was normalised using the norm1 approach where each variable is divided by the sum of all variables within a sample. In order to perform the PCA, the GC-MS variables were then mean centred and scaled to unit variance (autoscaling), while the NMR dataset was mean centred and pareto-scaled. Basically, autoscaling employs the standard deviation as a scaling factor thus giving all metabolites the same chance to affect the model, while pareto-scaling employs the square root of the standard deviation as scaling factor allowing the large fold changes to be less dominant compared to the original data^38^. Autoscaling is mainly used in metabolic profiling approach while mean-centring and pareto-scaling are preferred in case of metabolic fingerprinting in order to weigh down noisy signals and baseline noise^39^. PCA was then performed by using PLS Toolbox version 8.6.1 in Matlab (R2015b The Mathworks Inc., Natick, MA) environment. In order to assess if each metabolite’s concentration significantly differed among the studied groups, a one-way ANOVA (*anova1* function) test was performed. This test compares the means of two or more groups of data and returns the *p* values for the null hypothesis that the means of the groups are equal; however, it does not provide information about which pair of groups has statistically different levels of a certain metabolite. Thus, *multcompare* function, using the Tukey–Kramer correction, was employed to perform a multiple comparison (*post-hoc*) test. *Multcompare* routine uses the *anova1* results to determine which are the group means significantly different among all the possible pairwise combinations (CT-PH, CT-DS, CT-PD, PH-DS, PH-PD, and DS-PD). Thus, *p* values for all 85 variables of the combined (liver and colon) GC-MS datasets were calculated. *Anova1* and *multcompare* functions were available through a stats toolbox of Matlab (R2015b The Mathworks Inc., Natick, MA). A *p* value below .05 was considered statistically significant. Details about GC-MS metabolite identification, ANOVA, and multiple comparison analysis outputs are reported in Supplementary Table S2.

The impact of DSS and the phytosterol mixture on the metabolic pathways was evaluated by employing the pathway and set enrichment analysis tools of MetaboAnalyst (http://www.metaboanalyst.ca/MetaboAnalyst/faces/Home.jsp)^40^. The results are reported in Supplementary Figure S3.

## Results

The 40 mice employed in this study generated 4 groups: controls (CT), DSS-treated group (DS), PH, and the colitis phytosterol-treated group (PD). Liver and distal colons were recovered from each animal and they were both analysed by GC-MS, while only the liver samples (for quantitative issues) were further investigated by NMR.

### GC-MS metabolomics analysis

The GC-MS data included 53 variables for the liver and 32 variables for the colon samples. A univariate (one-way ANOVA) approach was employed to determine the statistically significant variables. Interestingly, 44 out of 53 variables were found to be statistically significant (*p* < .05) for liver and 25 out of 32 variables were found to be statistically significant for colon (Supplementary Table S2). Representative GC-MS chromatograms for colon and liver samples are reported in Supplementary Figure S2.

#### Distal colon

**Figure 2.**
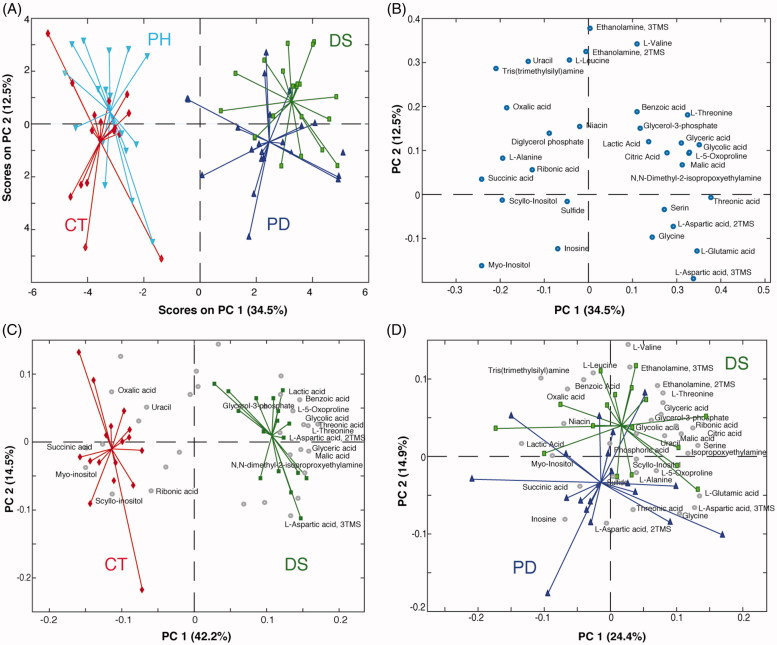
(A) PCA scores plot and (B) loading plot of the GC-MS data (distal colon). Bi-plot showing PC1 *vs.* PC2 scores of the PCA model calculated on (C) CT and DS groups and (D) DS and PD groups (distal colon). Keys: Controls (CT, red), DSS-induced colitis (DS, green), Phytosterols-fed colitis mice (PD, blue), and Phytosterols-fed control group (PH, light blue).

GC-MS data were submitted to multivariate data analysis to detect global correlations and differences among the samples. The initial matrix submitted to PCA consisted of 10 pooled and 72 real samples (8 out of 80 samples were discarded because of the poor quality of the chromatogram) and 32 variables. The pooled samples were used to monitor the instrument stability. Thus, after having verified that the pooled samples consistently lay in the very middle of the score plot, they were removed from the matrix and the PCA was recomputed. The score plot (Figure 2(A)) shows a very clear clustering of the studied groups along the Principal Component 1 (PC1), which accounts for the 34.5% of the total variance. In particular, the control group (CT) and the group of healthy mice treated with phytosterols (PH) are almost superimposed and lie in the left part of the plot opposed to groups of colitis mice (DS) and phytosterols-fed colitis mice (PD). PC2 does not contain information relative to the treatments, but it only shows the biological variability that characterise this animal model. The clear separation along PC1 confirms that the induction of the colitis definitively perturbs the whole metabolic system in the distal colon tissue^25^. Furthermore, the proximity of the phytosterol-fed groups to the control-diet ones (CT-PH and DS-PD) suggests that the effect of the phytosterols administration does not significantly affect the colon metabolome neither in healthy nor in colitis mice. The metabolites responsible for the groups’ separation are indicated in the loading plot (Figure 2(B)). In order to identify the potential markers related to the disease, a PCA only with data from control (CT) and colitis mice (DS) groups was computed (Figure 2(C)). Higher levels of small organic acids (lactic, benzoic, glycolic, threonic, malic, pyroglutamic, and glyceric), amino acids (serine, threonine, aspartic acid, and glycine) as well as a glycolysis precursor (glycerol-3-phosphate) were found in the inflamed colon of the DS mice. At the same time, control mice were characterised by higher concentrations of succinic, oxalic, and ribonic acids as well as scyllo-inositol, uracil, and myo-inositol.

It is also possible to identify the metabolites, and thus the metabolic pathways, that respond to the phytosterols treatment on colitis mice. This can be done computing a PCA including only DS and PD groups. As clearly shown in Figure 2(D), the PD mice are characterised by lower levels of amino acids (valine, leucine, threonine, and serine), small organic acids (malic, glyceric, ribonic, and citric), uracil, ethanolamine, and glycerol-3-phosphate in comparison to the DS ones.

#### Liver

**Figure 3.**
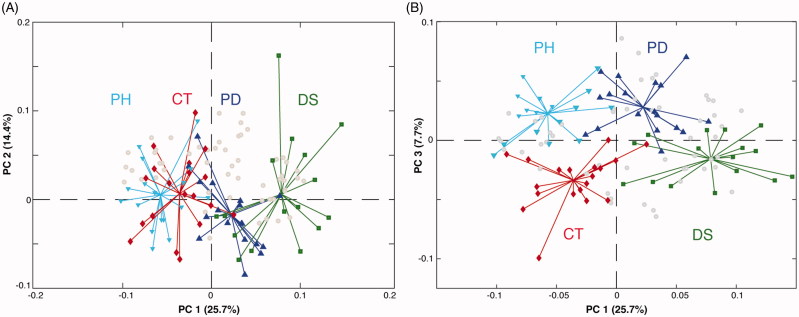
Bi-plot showing (A) PC1 *vs.* PC2 and (B) PC1 *vs.* PC3 scores and loadings of the PCA model calculated on all groups of the liver GC-MS data matrix. Keys: Controls (CT, red), DSS-induced colitis (DS, green), Phytosterols-fed colitis mice (PD, blue), and Phytosterols-fed control group (PH, light blue).

**Figure 4.**
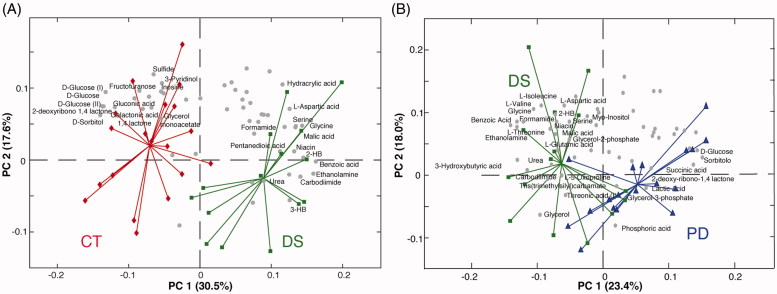
Bi-plots showing PC1 *vs.* PC2 scores and loadings of the PCA model calculated on (A) CT and DS groups and (B) DS and PD liver samples. Keys: Controls (CT, red), DSS-induced colitis (DS, green), Phytosterols-fed colitis mice (PD, blue), and Phytosterols-fed control group (PH, light blue).

The GC-MS data matrix obtained from liver samples consisted of 70 real samples (10 out of 80 samples were discarded because of the poor quality of the chromatogram) and 53 variables. Analogously to the colon analysis, 10 pooled samples were filtered out after checking the goodness of the instrument stability over time. Also in this case, the PCA provided interesting results (Figure 3(A)). PC1, which accounts for the 25.7% of the total variance, tends to separate the four groups. Thus, differently from what observed for the colon, where phytosterols-treated samples were almost superimposable to the phytosterols free ones, liver metabolism appears more susceptible to the administration of the sterols mixture employed. Indeed, a gradual shift of the metabolome from the colitis (DS) to the healthy (CT and PH) mice, passing through the phytosterol-treated (PD) group, is observed. Thus, the phytosterols seem to help to restore the biochemical equilibrium in the hepatic metabolome. As seen for the colon analysis, PC2 only explains the intra-group variability more than the inter-group variation. Interestingly, the computation of an additional PC3 (7.7% of variance explained), allowed us to detect a clear separation between the phytosterols-treated groups PH and PD and the control diet ones (CT and DS) (Figure 3(B)). This suggests that phytosterols administration slightly affects the hepatic metabolism independently by the presence/absence of the intestinal inflammation. In order to identify the effect of the disease on the liver metabolome, a PCA only with control (CT) and colitis mice (DS) groups was computed (Figure 4(A)). The concentration of three sugars (glucose, sorbitol, and fructose), as well as of gluconic acid and galactonic acid turned out to be higher in control mice, while six organic acids (malic, 3-hydroxybutyric, 2-hydroxybutyric, benzoic, hydracrylic, and pentanedioic), four amino acids (serine, glycine, L-threonine, and L-aspartic acid) as well as urea and niacin showed higher levels in the DS group. To better identify the metabolic pathway of the liver altered by the phytosterols treatment on colitis mice, a PCA including only DS and PD groups has been computed. The PCA bi-plot reported in Figure 4(B) indicates that the glucose, succinic acid, phosphoric acid, and glycerol-3-phosphate levels are higher in the PH, while the plant sterols help to reduce the high levels of 2-hydroxybutyrate (2-HB), 3-hydroxybutyrate (3-HB), and amino acids (valine, isoleucine, aspartic acid, glycine, threonine, and serine). Moreover, the ANOVA results reported in Supplementary Table S2 clearly shows that phytosterols administration significantly promotes the increase of glucose in colitis mice compared to the group that did not receive the diet supplement. Analogously, a significant reduced accumulation of 3-hydroxybutyric and 2-hydroxybutyric acid is observed in PD mice compared to the DS ones.

### NMR metabolomics analysis

**Figure 5.**
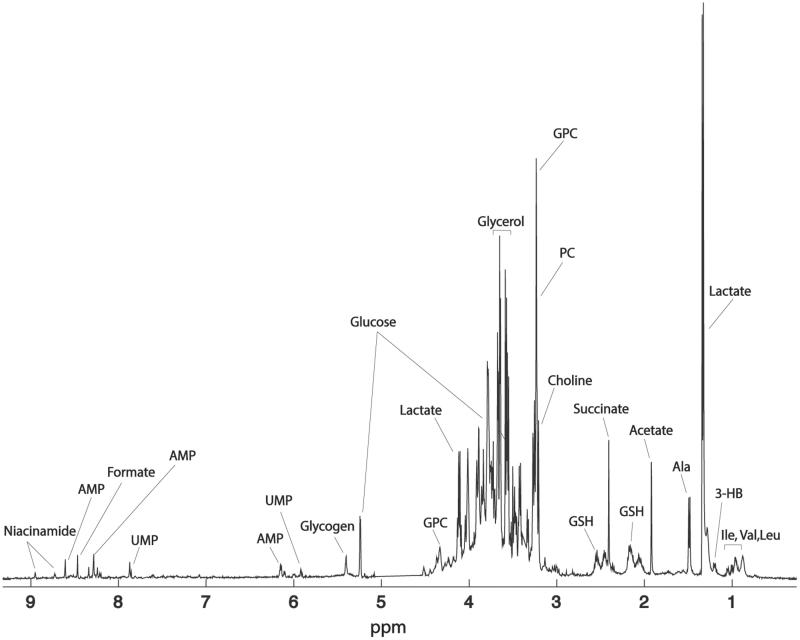
^1^H-NMR spectrum of a representative control sample along with the signal assignment. Keys: GPC: glycerophosphocholine; PC: phosphocholine; GSH: reduced glutathione; AMP: adenosine monophosphate; UMP: uridine monophosphate.

**Figure 6.**
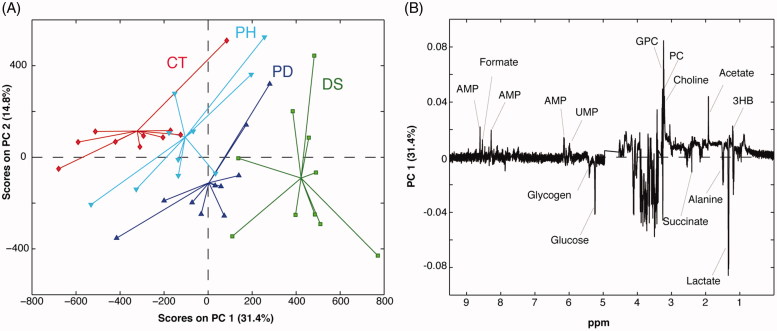
(A) PCA scores plot and (B) loading plot of the 1H-NMR spectra of mice liver extracts. Keys: Controls (CT, red), DSS-induced colitis (DS, green), Phytosterols-fed colitis mice (PD, blue), and Phytosterols-fed control group (PH, light blue).

**Figure 7.**
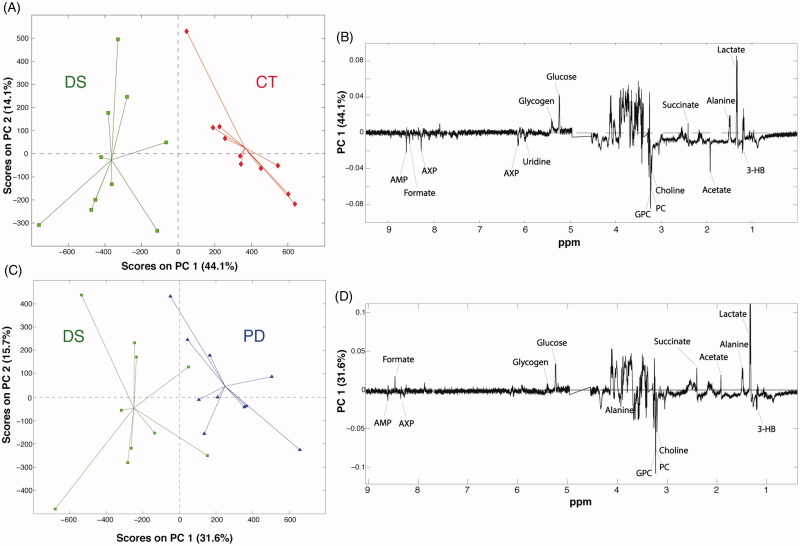
(A) PCA based on the pairwise comparison between controls (CT, red) and colitis group (DS, green), and (B) relative loading plot. (C) PCA based on the pairwise comparison between colitis mice fed with the phytosterols (PD, blue) and colitis group (DS, green), and (D) relative loading plot.

Given the interesting results obtained by the GC-MS analysis and the availability of the starting material, we decided to undertake also an NMR-based metabolomic study on the liver samples. Seventeen metabolites were identified in all the liver samples (Supplementary Table S3). The use of NMR allows detecting metabolites, such as the choline and its derivatives, as well as nucleotides, and nucleosides that are not observable by GC-MS. A representative NMR spectrum of the liver extracts is shown in Figure 5. Forty 1 D ^1^H-NMR spectra were processed and submitted to PCA. The PCA scores plot displaying the 2 main PCs explained 46.2% of the variance (PC-1 31.4%, PC-2 14.8%) (Figure 6). The PC1 loading plot shows a decrease in lactate, succinate, alanine, glucose, and glycogen levels in the liver samples belonging to the colitis (DS) and phytosterols-fed colitis mice (PD). At the same time, these groups showed higher levels of glycerophosphocholine (GPC), phosphocholine (PC), choline, acetate, 3-HB, adenosine monophosphate (AMP), formate, and uridine monophosphate (UMP). Interestingly, a clear trend that goes from the colitis mice group (DS) to the healthy one (CT) is visible along the PC1. In particular, livers extract of the phytosterol-treated colitis mice (PD) are situated exactly in the middle, between the controls and the colitis mice. This corroborates the GC-MS results described in the previous section, thus confirming the phytosterols ability to restore the homeostatic equilibrium of the hepatic metabolome. Two additional PCA were computed to allow a pairwise comparison of the studied groups. The first one was performed on healthy (CT) and DSS-treated (DS) samples (Figure 7(A)) to evaluate the perturbation caused by the DSS insult on the hepatic metabolome. The PC1 loading plot, reported in Figure 7(B), shows positive loading values for lactate, alanine, succinate, glucose, and glycogen thus indicating higher levels of such metabolites in control group compared to the DS one. On the other hand, an increase of 3-HB, acetate, choline, PC, GPC, UMP, AMP, and formate characterised the DSS-treated group. Then, in order to understand the effects of the phytosterols mixture on the DSS-induced perturbation, a PCA only including PD (phytosterols-fed colitis mice) and DS samples was performed (Figure 7(C)). Interestingly, the corresponding PC1 loading plot (Figure 7(D)) closely resembles the one obtained from the CT-DS comparison. This means that the phytosterols administration (PD group) is able to restore the homeostatic levels of lactate, alanine, succinate, glucose, glycogen, 3-HB, choline, PC, GPC, UMP, and AMP in the liver.

## Discussion

Phytosterols anti-inflammatory properties have recently been tested by Aldini et al. on an IBD mice model. They also found that the DSS-induced colitis was associated with body weight loss, visible faecal blood, diarrhoea, and other histopathological changes, such as ulcerations, polymorphonuclear cells, and mononuclear cells infiltration, together with a loss of epithelial structures. After DSS suspension, the remission phase turned out to be significantly accelerated in phytosterols-fed mice where incipient healing was observed. The authors concluded that, even if pre-treatment with phytosterols does not prevent the colitis onset, it definitively attenuates the morphologic damage while speeding up the mucosal healing during the remission phase^11^. In this study, we have performed a more in-depth study of the effects caused by the DSS insult and phytosterols administration at a metabolic level. In particular, we decided to employ an unsupervised approach, such as PCA, to explore the various datasets and to find changes in the metabolic pathways. Significant perturbations in carbohydrate, fatty acid, amino acids, and nucleotide metabolism as well as in the tricarboxylic acid (TCA) cycle were observed.

### Carbohydrate and fatty acid metabolism

It is well known that the intestinal damage caused by DSS severely alters the architecture of the colon mucosa. This leads to a reduced absorption of nutrients^25^ and subsequent changes in the energy metabolism. Thus, the organism tends to increase the glycolysis rate^25^^,^^35^ that is consistent with the observed reduction of glucose (NMR and GC-MS) and glycogen (NMR) levels in liver extracts of colitis mice. The up-regulation of the glycolysis process is also detectable from the increased concentration of lactate in the inflamed colonic mucosa cells^18^ that occurs in case of higher energy need and lack of energy sources under inflammatory conditions^41^. Interestingly, the phytosterols administration attenuated the DSS-related effects in the liver. In particular, higher glucose levels were detected in the liver of phytosterols-fed colitis mice (PD) livers compared to the untreated colitis mice (DS groups). The NMR liver analysis showed also a perturbation of phospholipid metabolism, in particular higher levels of choline and its derivatives (PC and GPC) were observed in DSS-treated mice in agreement with Dong et al. findings^25^. PC and GPC are involved in phospholipids metabolism which controls membrane’s assembly^42^. Hepatic accumulation of PC is associated with altered lipid metabolism occurring during the inflammation that characterises colitis mice. Recent studies found that DSS insult increases liver lipoprotein lipase expression and reduces both cytochrome P450 and acyl-CoA oxidase expression^28^^,^^43^. This is consistent with the higher levels of plasmatic cholesterol found by Aldini et al.^11^ as well as with the increased acetate concentration in livers of colitis mice compared to the controls. Indeed, acetate uptake increases when higher amount of acetyl-CoA, intermediate for synthesis of cholesterol and fatty acids, is needed^44^^,^^45^. Lower levels of PC and GPC were detected in phytosterols-fed colitis mice (PD) livers, showing the protective effects of the phytosterol administration. The activation of the fatty acid degradation as energy source is also consistent with the increase of the hepatic levels of 3-HB. 3-HB belongs to the ketonic bodies groups and an accumulation of these metabolites occurs when the lipids, and not the glucose, become the main source of energy for the organism. Moreover, higher levels of 2-HB were detected in the DS group (GC-MS). 2-HB is an early marker for impaired glucose regulation that appears to arise due to increased lipid oxidation and oxidative stress indeed; it generally appears at high concentrations case of deficient energy metabolism. Interestingly, a significant reduction in both 2- and 3-HB acids was observed in the phytosterols-fed colitis group compared to DS group, confirming, once again, the beneficial properties of the phytosterols mixture employed in this study. In addition, by comparing colitis mice (DS) and control (CT) groups, lower levels of myo-inositol were found in colitis colonic cells. Previous studies have hypothesised that extensive colonic inflammation and consequently a continuous high load of oxidative stress is correlated with low level of myo-inositol in the UC and it is associated with an increased risk of colorectal cancer^46^. A slight reduction of myo-inositol decrease was measured in PD (phytosterols-fed colitis) group compared to the DS (colitis mice) colon, suggesting the importance of antioxidant properties of phytosterols for UC healing, as previously reported^11^.

### Amino acid metabolism and TCA cycle

In agreement with the outcome of a recent GC-MS based metabolomic study conducted on sera and colon tissues of DSS-induced colitis mice^24^, we also detected higher levels of glycine, serine, and threonine in the inflamed colon of mice (DS) involved in our study. Threonine, glycine, and serine are extremely interconnected amino acids that belong to the same metabolic pathway as indicated also by the pathway analysis reported in Supplementary Figure S3. Indeed, Gu et al. found an up-regulation of the threonine aldolase gene expression in colitis mice. This enzyme catalyses the reaction that produces glycine and acetaldehyde starting from the L-threonine. Jain et al. reported a correlation between glycine and a disorder in the glycolysis process, thus representing a key metabolite in the rapid cell proliferation of tumors^47^. Moreover, serine is derived from glycerol-3-phosphate, a glycolysis intermediate whose level increased in colitis mice^48^. In addition, recent studies showed that serine and glycine biosynthesis might suppress cellular antioxidative capacities and thus supported colon tumour homeostasis^49^. Interestingly, the phytosterols-fed (PD) group showed decreased colonic levels of serine and threonine compared to the DS group, while no significant changes in glycine concentration was appreciated. The correlation between UC and Krebs cycle-related molecules was recently investigated by Azuma et al.^19^. Six intermediates (citric, fumaric, isocitric, malic, pyruvic, and succinic acid) were found to be increased in UC patients compared to the healthy individuals. In agreement with the cited study, we also found higher levels of malic and citric acids in the inflamed colons compared to the control group. Interestingly, phytosterols were able to reduce the up-regulation of the Krebs cycle induced by the DSS insult, since lower levels of both acids were detected in the PD mice compared to the DS group.

### Nucleotide (purine and pyrimidine) metabolism

Interestingly, liver extracts from mice with DSS-induced colitis showed some dysfunctions in the nucleic acid synthesis/degradation pathways since adenosine 5′-monophosphate, adenosine 5′-triphosphate, and UMP levels were higher in colitis mice in agreement with data reported in literature^25^. Since the liver is the primary organ for nucleotide synthesis, our observation implies colitis may cause the degradation of nucleic acids or inhibit the synthesis of DNA and RNA, as these metabolites are the basic structural units of nucleic acids. A restored equilibrium, in this particular pathway, seems to be generated in the PD group compared to the DSS-treated mice, proving once again the healthy effect of phytosterol administration on liver homeostasis.

## Conclusions

In this exploratory study, a combined GC-MS and NMR-based metabolomics approach was used to provide further insights into the phytosterols role in prevention and remission of DSS-induced colitis. In particular, an unsupervised data analysis approach was employed to identify the metabolic pathways perturbed by the colitis and restored by the phytosterols. Indeed, by means of PCA, we detected a healing trend for the phytosterols-fed mice, for which the homeostatic equilibrium in key metabolic pathways, such as glycolysis, Krebs cycle, amino acids, and nucleotide metabolism seems to be re-established. These findings suggest that the phytosterols mixture employed in this work may be taken into consideration as potential nutraceutical tool in the treatment and prevention of IBDs, thus reducing their growing impact on the population.

## Supplementary Material

Supplemental Material
