# Supplementary material for: Impact of phytosterols on liver and distal colon metabolome in experimental murine colitis model: an explorative study
Source: J Enzyme Inhib Med Chem. 2019 May 10;34(1):1041–50. doi: 10.1080/14756366.2019.1611802 (PMC6522980; doi:10.1080/14756366.2019.1611802)
Supplement: Supplemental Material [file IENZ_A_1611802_SM8107.pdf]

Supplementary Information

Impact of phytosterols on liver and distal colon metabolome in experimental murine colitis model

Nunzia Iaccarino[1], Jussara Amato[1,*], Bruno Pagano[1], Anna Di Porzio[1], Ettore Novellino[1], Matteo Micucci[2], Luca Bolelli[2], Rita Aldini[2], Roberta Budriesi[2], Antonio Randazzo[1,*]

[1]Department of Pharmacy, University of Naples Federico II, 80131 Naples, Italy.

[2]Department of Pharmacy and Biotechnology, University of Bologna, 40126 Bologna, Italy.

Table of Contents

	Content	Page
1	**Figure S1.** Chemical structure of the administered phytosterols.	S3
2	**Figure S2.** Representative GC-MS chromatograms obtained from the metabolome analysis of (A) liver and (B) distal colon.	S4
3	**Figure S3.** Pathway analysis of (A) colon and (C) liver extracts and metabolite set enrichment overview of (B) colon and (D) liver extracts.	S5
4	**Table S1.** Composition of phytosterols preparation.	S6
5	**Table S2.** List of all metabolites identified in liver and colon extracts by GC-MS. Full name, short name, RT, RI, RI(NIST), ΔRI, ID level, CAS registry number and *p*-value from one-way ANOVA are indicated for each compound.	S7
6	**Table S3.** List of all metabolites identified in liver samples by NMR.	S9

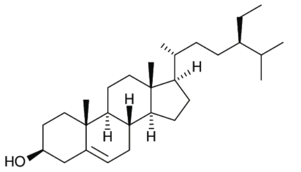

β-sitosterol

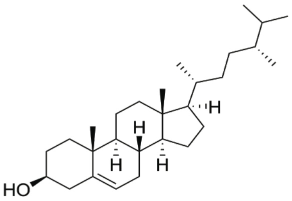

Campesterol

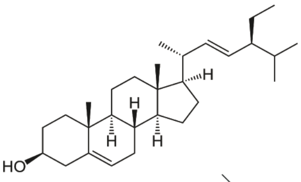

Stigmasterol

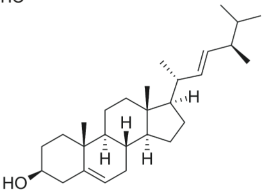

Brassicasterol

Figure S1. Chemical structure of the administered phytosterols.

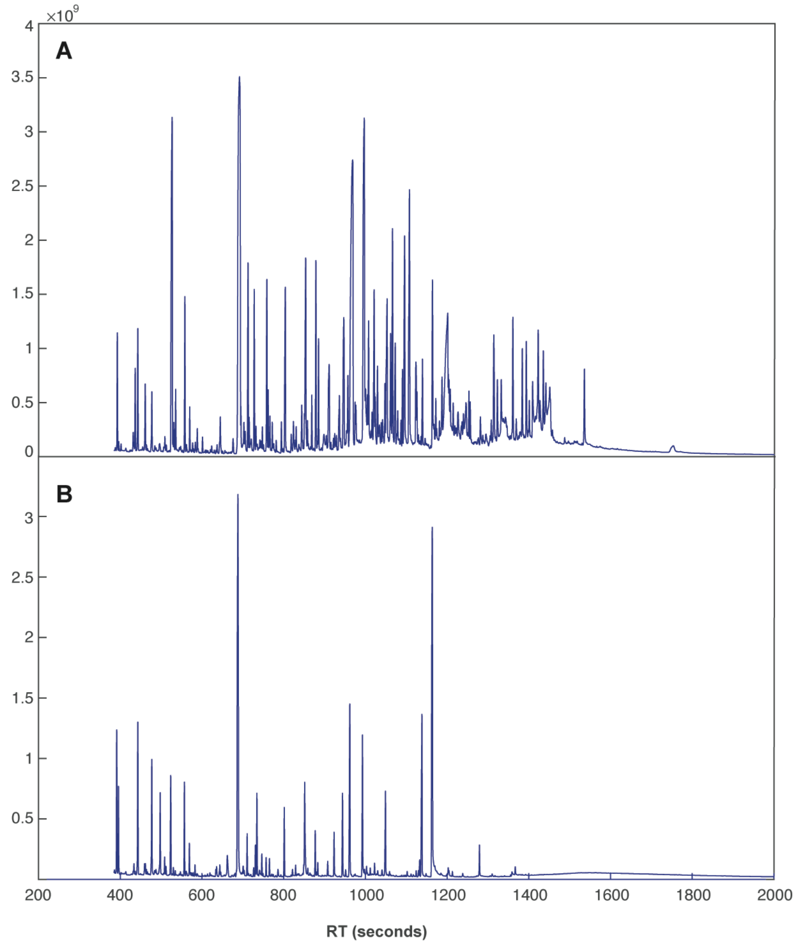

Figure S2. Representative GC-MS chromatograms obtained from the metabolome analysis of (A) liver and (B) distal colon.

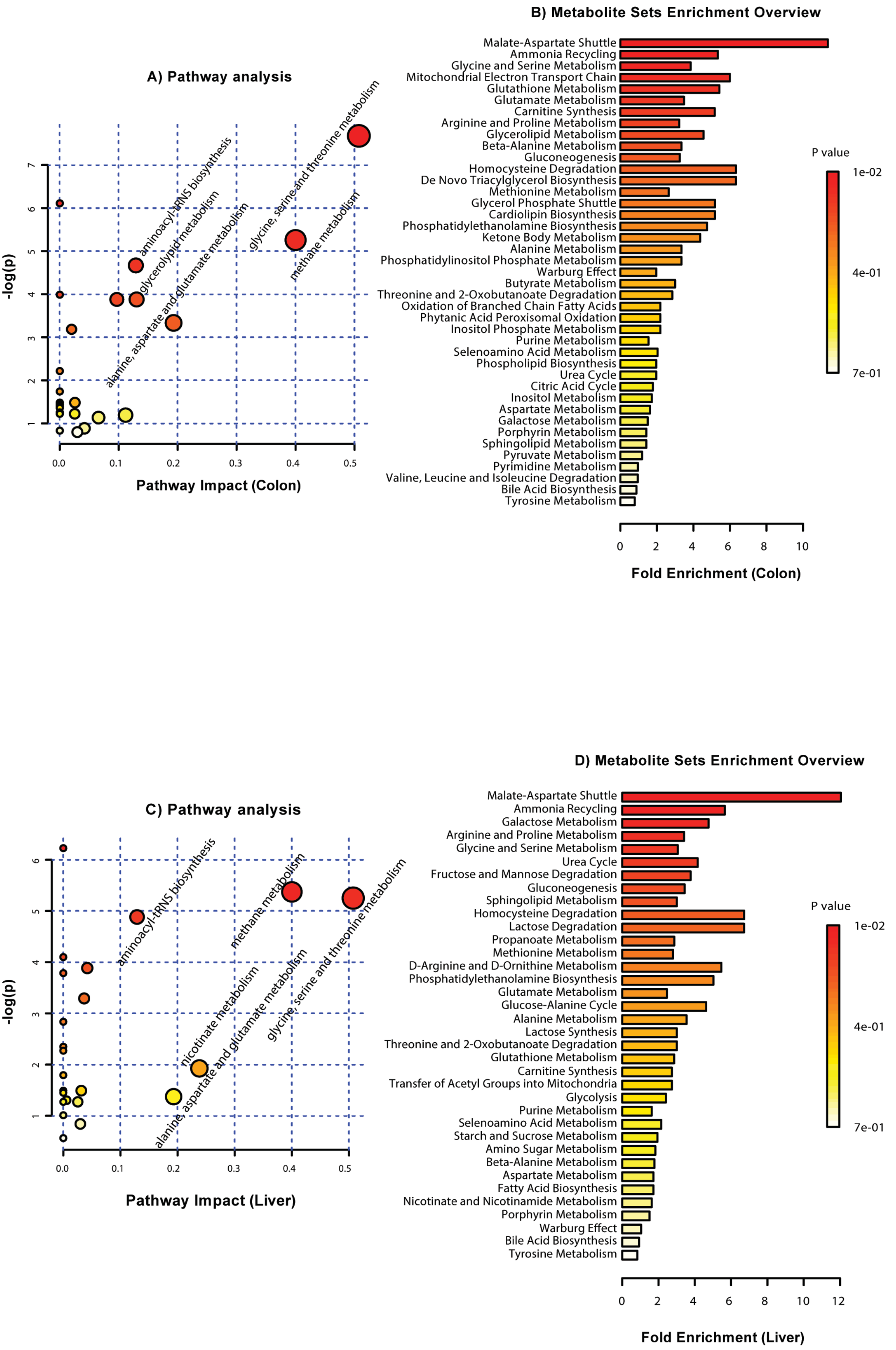

Figure S3. Pathway analysis of (A) colon and (C) liver extracts and metabolite set enrichment overview of (B) colon and (D) liver extracts.

Table S1. Composition of Phytosterols preparation.

**Ingredient**	**Quantity (mg/4 pearls)**
Mixture of phytosterols (from soy)	2000 mg
Total plant sterol esters	1800 mg
Edible gelatin	931 mg
Agent of resistance	
Glycerol	429 mg
Carthamus Tinctorius (Safflower) Seed Oil	180 mg
Dye	
Caramel	68 mg

Table S2. List of all metabolites identified in liver and colon extracts by GC-MS. Full name, short name, RT, RI, RI(NIST), ΔRI, ID level, CAS registry number and *p*-value from one-way ANOVA are indicated for each compound.

ID #	Metabolite full name from NIST[a]	Metabolite short name[b]	RT (s)[c]	RI[d]	RI (NIST)[e]	ΔRI[f]	ID[g]	CAS[h]	One-way ANOVA[i]					
									**CT-PH**	**CT-DS**	**CT-PD**	**PH-DS**	**PH-PD**	**DS-PD**
	Liver													
1	Formamide, 2TMS derivative	Formamide, 2TMS	431.2	955.2	948	-7.2	2	15500-60-4	N.S.	3.17E-02	N.S.	8.42E-03	3.29E-02	N.S.
2	Disilathiane, hexamethyl-	Sulfide, 2TMS	435.9	961.1	963.1	2	2	3385-94-2	N.S.	1.95E-05	N.S.	2.34E-05	N.S.	N.S.
3	Silanamine, N,N -methanetetraylbis[1,1,1-trimethyl-	Carbodiimide, 2TMS	442.7	969.6	964	-5.6	2	1000-70-0	N.S.	3.40E-04	N.S.	7.74E-04	N.S.	N.S.
4	Ethanolamine, 2TMS derivative	Ethanolamine, 2TMS	497.4	1037.9	1020.8	-17.1	2	17165-52-5	N.S.	3.77E-09	N.S.	3.78E-09	N.S.	3.79E-08
5	Lactic Acid, 2TMS derivative	Lactic Acid, 2TMS	524.6	1071.8	1086	14.2	2	17596-96-2	N.S.	N.S.	N.S.	N.S.	N.S.	N.S.
6	Glycolic acid, 2TMS derivative	Glycolic acid, 2TMS	535	1084.9	1070.5	-14.4	2	33581-77-0	5.58E-03	N.S.	1.95E-03	N.S.	N.S.	3.50E-02
7	L-Alanine, 2TMS derivative	L-Alanine, 2TMS	557.6	1113.1	1105.3	-7.8	2	27844-07-1	4.49E-02	N.S.	N.S.	6.84E-03	7.36E-05	N.S.
8	Glycine, di-TMS	Glycine, 2TMS	569.2	1127.6	1121.5	-6.1	2	7364-42-3	2.10E-05	1.04E-05	N.S.	3.77E-09	1.20E-03	2.14E-07
9	2-Hydroxybutyric acid, 2TMS derivative	2-Hydroxybutyric acid, 2TMS	576.2	1136.4	1136	-0.4	2	55133-93-2	N.S.	4.87E-09	4.71E-06	2.21E-05	2.24E-02	N.S.
10	3-Pyridinol, TMS derivative	3-Pyridinol, TMS	581.5	1143	1134.1	-8.9	2	41571-88-4	8.47E-03	1.27E-02	N.S.	1.00E+00	N.S.	N.S.
11	Hydracrylic acid, 2TMS derivative	Hydracrylic acid, 2TMS	587.7	1150.7	1151	0.3	2	55162-32-8	N.S.	3.98E-02	1.47E-02	8.47E-01	N.S.	N.S.
12	3-Hydroxybutyric acid, 2TMS derivative	3-Hydroxybutyric acid, 2TMS	600.4	1166.6	1167	0.4	2	55133-94-3	N.S.	3.77E-09	N.S.	3.79E-09	N.S.	3.86E-09
13	Tris(trimethylsilyl)carbamate	Carbamate, 3TMS	633.7	1203.5	962	-241.5	3	91719351*	N.S.	7.15E-03	4.94E-02	N.S.	N.S.	N.S.
14	L-Valine, 2TMS derivative	L-Valine, 2TMS	643.8	1208.9	1208.4	-0.5	2	7364-44-5	4.15E-03	N.S.	6.77E-06	7.90E-04	N.S.	1.22E-06
15	Benzoic Acid, TMS derivative	Benzoic Acid, TMS	664.2	1219.8	1242	22.2	2	2078-12-8	N.S.	4.14E-09	1.15E-03	3.60E-07	N.S.	1.17E-03
16	Urea, 2TMS derivative	Urea, 2TMS	669.1	1222.4	1237	14.6	2	18297-63-7	N.S.	3.34E-04	N.S.	6.21E-04	N.S.	1.69E-02
17	Glycerol, 3TMS derivative	Glycerol, 3TMS	691.1	1234.1	1265.1	31	2	6787-10-6	N.S.	N.S.	N.S.	1.15E-03	N.S.	N.S.
18	Silanol, trimethyl-, phosphate (3:1)	Phosphoric acid, 3TMS	692.4	1234.8	1263	28.2	2	10497-05-9	N.S.	N.S.	N.S.	N.S.	N.S.	N.S.
19	Niacin, TMS derivative	Niacin, TMS	698.2	1238	1257	19	2	25436-37-7	N.S.	2.09E-06	N.S.	2.37E-08	N.S.	9.74E-05
20	L-Isoleucine, 2TMS derivative	L-Isoleucine, 2TMS	701.5	1239.7	1286.2	46.5	3	7483-92-3	2.06E-03	N.S.	3.33E-06	5.74E-04	N.S.	9.81E-07
21	L-Proline, 2TMS derivative	L-Proline, 2TMS	704.6	1241.4	1304.9	63.5	3	7364-47-8	1.21E-04	N.S.	6.59E-04	N.S.	N.S.	N.S.
22	Butanedioic acid, 2TMS derivative	Succinic acid, 2TMS	711.9	1245.3	1314.4	69.1	3	40309-57-7	N.S.	N.S.	1.19E-03	N.S.	6.11E-03	2.58E-04
23	1-Monoacetin, 2O-TMS	Glycerol monoacetate, 2TMS	714.5	1246.6	1323.7	77.1	3	-	N.S.	3.59E-02	6.20E-03	N.S.	N.S.	N.S.
24	Glyceric acid, 3TMS derivative	Glyceric acid, 3TMS	726.9	1253.3	1199	-54.3	3	38191-87-6	N.S.	N.S.	N.S.	N.S.	N.S.	N.S.
25	Uracil, 2TMS derivative	Uracil, 2TMS	731.1	1255.5	1331.9	76.4	3	10457-14-4	N.S.	N.S.	N.S.	N.S.	N.S.	N.S.
26	Serine, 3TMS derivative	Serine, 3TMS	746.7	1263.9	1342.6	78.7	3	64625-17-8	N.S.	3.14E-05	2.37E-02	6.93E-04	N.S.	N.S.
27	L-Threonine, 3TMS derivative	L-Threonine, 3TMS	765.2	1273.8	1367.4	93.6	3	7537-02-2	N.S.	N.S.	N.S.	7.26E-05	N.S.	7.11E-03
28	Pentanedioic acid, 2TMS derivative	Pentanedioic acid, 2TMS	771	1276.9	1394.5	117.6	3	55494-07-0	3.38E-07	8.81E-03	4.34E-03	4.53E-02	N.S.	N.S.
29	Butanoic acid, 3,4-bis[(trimethylsilyl)oxy]-, trimethylsilyl ester	2-Deoxytetronic acid, 3TMS	793.3	1288.8	1417.3	128.5	3	55191-53-2	N.S.	N.S.	N.S.	N.S.	N.S.	N.S.
30	Niacinamide, TMS derivative	Niacinamide, TMS	823	1304.7	2041	736.3	3	91727260*	N.S.	N.S.	N.S.	N.S.	N.S.	N.S.
31	Malic acid, 3TMS derivative	Malic acid, 3TMS	829.4	1308.1	1496.9	188.8	3	38166-11-9	N.S.	1.25E-05	1.47E-04	1.62E-05	1.80E-04	N.S.
32	4-Hydroxy-5-(hydroxymethyl)oxolan-2-one, 2TMS derivative	2-deoxy-ribono-1,4-lactone,2TMS	843	1315.3	1416	100.7	3	-	3.55E-02	1.23E-05	N.S.	4.26E-09	1.97E-04	5.24E-03
33	L-Aspartic acid, 3TMS derivative	L-Aspartic acid, 3TMS	849.6	1318.9	1502.4	183.5	3	55268-53-6	N.S.	6.00E-04	N.S.	1.99E-06	9.86E-04	N.S.
34	L-5-Oxoproline, 2TMS derivative	L-5-Oxoproline, 2TMS	852.8	1320.6	1520.7	200.1	3	30274-77-2	2.05E-02	N.S.	N.S.	3.14E-03	1.09E-02	N.S.
35	2,3,4-Trihydroxybutyric acid tetrakis(trimethylsilyl) deriv., (, (R*,R*)-)	Threonic acid, 4TMS	867.8	1328.5	1518	189.5	3	528672*	N.S.	N.S.	N.S.	N.S.	N.S.	N.S.
36	2,3,4-Trihydroxybutyric acid tetrakis(trimethylsilyl) deriv., (, (R*,R*)-) (I)	Threonic acid, 4TMS (I)	877.5	1333.8	1518	184.2	3	528672*	N.S.	N.S.	N.S.	4.85E-03	1.04E-02	N.S.
37	L-Glutamic acid, 3TMS derivative	L-Glutamic acid, 3TMS	908.1	1350.1	1629.4	279.3	3	15985-07-6	N.S.	N.S.	N.S.	9.81E-03	3.20E-02	N.S.
38	2,3,4,5-Tetrahydroxypentanoic acid-1,4-lactone, tris(trimethylsilyl)-	Arabinonic acid, 1,4-lactone	920.6	1356.8	1662	305.2	3	74742-31-7	3.93E-02	N.S.	N.S.	1.18E-04	1.71E-03	N.S.
39	Phosphoric acid, 2-(trimethylsiloxy)-1-[(trimethylsiloxy)methyl]ethyl bis(trimethylsilyl) ester	Glycerol-2-phosphate, 4TMS	973.7	1385.2	1708.5	323.3	3	31038-12-7	N.S.	N.S.	2.43E-03	N.S.	N.S.	N.S.
40	Phosphoric acid, bis(trimethylsilyl) 2,3-bis[(trimethylsilyl)oxy]propyl ester	Glycerol-3-phosphate, 4TMS	996.9	1397.5	1787	389.5	3	31038-11-6	N.S.	N.S.	N.S.	N.S.	N.S.	N.S.
41	Ribonic acid, 2,3,4,5-tetrakis-O-(trimethylsilyl)-, trimethylsilyl ester	Ribonic acid, 5TMS	1006.9	1910.1	1799	-111.1	3	57197-35-0	N.S.	N.S.	N.S.	N.S.	N.S.	N.S.

42	D-(-)-Fructofuranose, pentakis(trimethylsilyl) ether (isomer 1)	Fructofuranose, 5TMS	1022.9	1925.2	1792.1	-133.1	3	-	N.S.	5.91E-04	1.80E-05	6.03E-03	3.00E-04	N.S.
43	D-Glucose, 5TMS derivative	D-Glucose, 5TMS	1064.9	1964.9	1926	-38.9	2	6736-97-6	N.S.	3.02E-07	3.06E-03	3.86E-09	1.59E-06	N.S.
44	D-Glucose, 5TMS derivative (I)	D-Glucose, 5TMS (I)	1071.8	1971.4	1926	-45.4	3	6736-97-6	N.S.	1.52E-08	2.52E-06	7.11E-08	1.25E-05	N.S.
45	L-Glucono-1,4-lactone (2S,3R,4S,5S)-, 4TMS derivative	Galactonic acid 1,4-lactone, 4TMS	1077.8	1977.1	1981	3.9	2	523382*	N.S.	5.39E-03	3.74E-03	1.14E-05	6.07E-06	N.S.
46	D-Sorbitol, hexakis(trimethylsilyl) ether	Sorbitol, 6TMS	1089.3	1987.9	1988	0.1	2	91746474*	N.S.	4.78E-07	9.71E-04	8.91E-09	1.31E-05	N.S.
47	D-Glucose, 5TMS derivative (II)	D-Glucose, 5TMS (II)	1106.5	2009.6	1926	-83.6	3	6736-97-6	N.S.	6.81E-06	2.62E-04	3.50E-05	1.11E-03	N.S.
48	D-Gluconic acid, 6TMS derivative	D-Gluconic acid, 6TMS	1128.4	2057.4	2043	-14.4	2	34290-52-3	N.S.	2.39E-03	N.S.	2.06E-04	N.S.	N.S.
49	Myo-Inositol, 6TMS derivative	Myo-Inositol, 6TMS	1163.1	2132.9	2130	-2.9	2	2582-79-8	N.S.	N.S.	N.S.	N.S.	N.S.	N.S.
50	Glyceryl-glycoside TMS ether	Glyceryl-glycoside TMS ether	1251.8	2337.7	2376	38.3	3	91733362*	6.38E-03	N.S.	N.S.	4.39E-04	2.35E-04	N.S.
51	Glyceryl-glycoside TMS ether (II)	Glyceryl-glycoside TMS ether	1255	2345.3	2376	30.7	2	91733362*	N.S.	N.S.	N.S.	4.78E-03	6.48E-03	N.S.
52	Inosine, 4TMS derivative	Inosine, 4TMS	1359.7	2610.6	2564.4	-46.2	3	6428469*	N.S.	1.50E-03	2.17E-02	8.79E-03	N.S.	N.S.
53	Adenosine, 4TMS derivative	Adenosine, 4TMS	1383	2675.1	2625.8	-49.3	3	53294-33-0	N.S.	N.S.	N.S.	N.S.	N.S.	N.S.
	Colon													
1	Disilathiane, hexamethyl-	Sulfide, 2TMS	436.1	961.3	963.1	1.8	2	3385-94-2	N.S.	3.77E-09	3.77E-09	3.77E-09	3.77E-09	1.34E-02
2	Ethanolamine, 2TMS derivative	Ethanolamine, 2TMS	497.6	1038.1	1020.8	-17.3	2	17165-52-5	1.41E-08	4.07E-09	9.17E-06	N.S.	N.S.	3.74E-02
3	Lactic Acid, 2TMS derivative	Lactic Acid, 2TMS	524.3	1071.5	1.086	14.5	2	17596-96-2	N.S.	3.77E-09	1.59E-08	3.77E-09	7.11E-07	2.51E-03
4	Glycolic acid, 2TMS derivative	Glycolic acid, 2TMS	534.7	1084.3	1070.5	-13.8	2	33581-77-0	N.S.	1.64E-06	N.S.	2.43E-05	N.S.	3.72E-04
5	L-Alanine, 2TMS derivative	L-Alanine, 2TMS	557	1112.3	1105.3	-7	2	27844-07-1	N.S.	N.S.	N.S.	N.S.	N.S.	N.S.
6	Glycine, di-TMS	Glycine, 2TMS	569.1	1127.5	1121.5	-6	2	7364-42-3	N.S.	N.S.	N.S.	N.S.	N.S.	N.S.
7	Tris(trimethylsilyl)amine	Tris(trimethylsilyl)amine	570.3	1128.9	695	-433.9	3	1586-73-8	N.S.	N.S.	N.S.	N.S.	N.S.	N.S.
8	Oxalic acid, 2TMS derivative	Oxalic acid, 2TMS	578.4	1139.1	1.122	-17.1	2	18294-04-7	N.S.	3.77E-09	1.63E-02	3.78E-09	N.S.	1.15E-08
9	L-Valine, 2TMS derivative	L-Valine, 2TMS	643.7	1208.9	1208.4	-0.5	2	7364-44-5	N.S.	N.S.	3.24E-02	N.S.	N.S.	N.S.
10	Benzoic Acid, TMS derivative	Benzoic Acid, TMS	664.3	1219.8	1.242	22.2	2	2078-12-8	N.S.	3.77E-09	3.77E-09	3.77E-09	3.98E-09	1.07E-02
11	Ethanolamine, 3TMS derivative	Ethanolamine, 3TMS	681	1228.8	1257.4	28.6	2	5630-81-9	N.S.	N.S.	N.S.	N.S.	N.S.	N.S.
12	L-Leucine, 2TMS derivative	L-Leucine, 2TMS	684.1	1230.4	1276.4	46	3	7364-46-7	N.S.	3.00E-06	5.60E-09	2.26E-08	3.78E-09	N.S.
13	Niacin, TMS derivative	Niacin, TMS	698.0	1237.9	1.257	19.1	2	25436-37-7	N.S.	3.85E-09	6.99E-06	1.15E-08	4.56E-04	1.96E-02
14	Butanedioic acid, 2TMS derivative	Succinic acid, 2TMS	710.6	1244.6	1314.4	69.8	3	40309-57-7	N.S.	3.67E-03	1.58E-07	6.35E-04	1.75E-08	3.18E-02
15	Glyceric acid, 3TMS derivative	Glyceric acid, 3TMS	726.4	1.253	1318.4	65.4	3	38191-87-6	N.S.	2.87E-04	9.53E-06	2.70E-04	8.37E-06	N.S.
16	Uracil, 2TMS derivative	Uracil, 2TMS	730.8	1255.4	1331.9	76.5	3	10457-14-4	N.S.	1.64E-04	3.53E-08	1.06E-05	4.99E-09	N.S.
17	Serine, 3TMS derivative	Serine, 3TMS	746.5	1263.8	1342.6	78.8	3	64625-17-8	N.S.	N.S.	N.S.	N.S.	N.S.	N.S.
18	L-Threonine, 3TMS derivative	L-Threonine, 3TMS	765.2	1273.8	1367.4	93.6	3	7537-02-2	N.S.	1.03E-08	1.02E-06	6.12E-07	8.16E-05	N.S.
19	L-Aspartic acid, 2TMS derivative	L-Aspartic acid, 2TMS	786.7	1285.2	1.427	141.8	3	8583068*	N.S.	1.27E-04	N.S.	5.77E-03	N.S.	5.96E-03
20	Malic acid, 3TMS derivative	Malic acid, 3TMS	829.2	1.308	1496.9	188.9	3	38166-11-9	N.S.	5.29E-05	2.06E-07	3.94E-02	5.28E-04	N.S.
21	L-Aspartic acid, 3TMS derivative	L-Aspartic acid, 3TMS	849.2	1318.6	1502.4	183.8	3	55268-53-6	N.S.	3.78E-09	1.04E-03	3.93E-09	8.61E-03	3.60E-05
22	L-5-Oxoproline, 2TMS derivative	L-5-Oxoproline, 2TMS	851.4	1319.8	1520.7	200.9	3	30274-77-2	N.S.	3.93E-09	3.77E-09	6.07E-08	4.05E-09	N.S.
23	2,3,4-Trihydroxybutyric acid tetrakis(trimethylsilyl) deriv., (, (R*,R*)-)	Threonic acid, 4TMS	876.9	1333.4	1.518	184.6	3	528672*	N.S.	2.06E-05	4.48E-03	4.24E-06	1.29E-03	N.S.
24	L-Glutamic acid, 3TMS derivative	L-Glutamic acid, 3TMS	907.7	1349.9	1629.4	279.5	3	15985-07-6	N.S.	N.S.	N.S.	N.S.	N.S.	N.S.
25	Phosphoric acid, bis(trimethylsilyl) 2,3-bis[(trimethylsilyl)oxy]propyl ester	Glycerol-3-phosphate, 4TMS	992.7	1395.3	1743.1	347.8	3	31038-11-6	N.S.	9.44E-04	9.12E-04	7.67E-03	7.60E-03	N.S.
26	Ribonic acid, 2,3,4,5-tetrakis-O-(trimethylsilyl)-, trimethylsilyl ester	Ribonic acid, 5TMS	997.7	1.398	1.799	401	3	57197-35-0	N.S.	3.42E-02	N.S.	1.39E-02	N.S.	N.S.
27	Citric acid, 4TMS derivative	Citric acid, 4TMS	1022.2	1924.6	1.944	19.4	2	14330-97-3	N.S.	N.S.	N.S.	N.S.	N.S.	N.S.
28	N,N-Dimethyl-2-isopropoxyethylamine	N,N-Dimethyl-2-isopropoxyethylamine	1059.1	1959.4	778	-1181.4	3	71126-59-5	N.S.	3.96E-02	2.31E-06	7.23E-03	1.60E-07	1.96E-02
29	Scyllo-Inositol, 6TMS derivative	Scyllo-Inositol, 6TMS	1132.7	2066.6	2.194	127.4	3	2582-79-8	2.47E-05	1.40E-05	1.15E-08	3.77E-09	3.77E-09	N.S.
30	Myo-Inositol, 6TMS derivative	Myo-Inositol, 6TMS	1163.6	2.134	2.194	60	3	2582-79-8	N.S.	3.40E-02	N.S.	2.34E-02	2.01E-01	N.S.
31	Phosphoric acid, bis[2,3-bis[(trimethylsilyl)oxy]propyl] trimethylsilyl ester	Diglycerol phosphate	1204.7	2225.7	2.182	-43.7	3	32046-28-9	N.S.	5.26E-03	1.66E-03	6.13E-04	1.63E-04	N.S.
32	Inosine, 4TMS derivative	Inosine, 4TMS	1360.1	2611.7	2564.4	-47.3	3	6428469*	N.S.	N.S.	N.S.	3.59E-04	9.78E-04	N.S.

[a]Full names of metabolites are directly extracted from the NIST11 metabolite database. [b]Metabolite short names are IUPAC or generic names of the identified metabolites. [c]Retention times of deconvoluted peaks were calculated as a mean of RTs of each peak across all samples. [d]Based on these RTs, retention indices (RI) of metabolites were estimated using all even alkane mixture sample (C10-C40). [e]Reported RIs of identified metabolites were extracted from NIST 11 *version 2.3*. [f]Difference between the reported RI and the calculated RI. [g]Identification level of the metabolites. Level 1 if the peaks are confirmed using authentic standards; Level 2 when the peaks are identified based on their EI-MS ≥ 80 (%) and RI match (±30); Level 3 when the peaks are identified based only on their EI-MS ≥ 80 (%) and the RI match is larger than 30. [h]CAS number of identified metabolites. For the metabolites with no CAS number the PubChem CID number has been reported followed by a (*). [i]One-way ANOVA test were performed to see if there are some variables significantly different among the groups: control (CT), control fed with phytosterols (PH), DSS treated (DS), DSS-treated fed with phytosterols (PD). Significance of effects within the different pairs of groups is evaluated by *P*-values. "N.S." = Not Significant (P>0.05).

Table S3. NMR assignment of the identified metabolites.

Identification number	Metabolite	Chemical Shifts (ppm)
**1**	Isoleucine, Leucine, Valine	0.94(t)<br>1.02(d)<br>0.97(d)<br>0.99(d)<br>1.05(d)
**2**	3-hydroxybutyrate, 3-HB	1.20(d)<br>2.31(dd)
**3**	Lactate	1.33(d)<br>4.13(q)
**4**	Alanine	1.49(d)<br>3.81(q)
**5**	Acetate	1.92(s)
**6**	Glutathione, GSH	2.54(m)<br>2.16(m)
**7**	Succinate	2.40(s)
**8**	Choline	3.20(s)
**9**	O-Phosphocholine, PC	3.23(s)<br>4.17(m)
**10**	Glycerophosphocholine, GPC	3.23(s)<br>3.62(m)<br>4.33(m)
**11**	Glycerol	3.56(m)<br>3.66(m)<br>3.78(m)
**12**	Glucose	3.39(m)<br>3.46(m)<br>3.53(m)<br>3.73(m)<br>3.84(m)<br>3.90(dd)<br>5.24(d)
**13**	Glycogen	5.40(br s)
**14**	Uridine monophosphate, UMP	4.36(t)<br>5.98(m)<br>8.10(d)
**15**	Adenosine monophosphate, AMP	4.02(dd)<br>4.36(dd)<br>4.51(dd)<br>6.14(d)<br>8.24(s)<br>8.59(s)
**16**	Formate	8.44(s)
**17**	Niacinamide	7.58(dd)<br>8.24(dd)<br>8.70(dd)<br>8.92(s)
